# Congestive heart failure is the leading cause of pleural effusion in the north-west of Iran

**DOI:** 10.15171/jcvtr.2019.40

**Published:** 2019-08-01

**Authors:** Masoud Nazemiyeh, Amirhossein Dorraji, Masoud Nouri-Vaskeh, Akbar Sharifi

**Affiliations:** ^1^Tuberculosis and Lung Diseases Research Center, Tabriz University of Medical Sciences, Tabriz, Iran; ^2^Connective Tissue Diseases Research Center, Tabriz University of Medical Sciences, Tabriz, Iran

**Keywords:** Pleural Effusion, Heart Failure, Exudates and Transudates, Tuberculosis

## Abstract

***Introduction:*** Pleural effusion (PE) is a common manifestation of pulmonary and non–pulmonary diseases, and the first step for diagnosing the etiology is analysis of pleural fluid. The aim of this study was to determine the epidemiology of PE in a tertiary referral hospital in the North-West of Iran.

***Methods:*** All patients with PE who referred to the department of pulmonary diseases in tertiary centre of Tabriz University of Medical Sciences between 2015 and 2016 were enrolled. Complete enumeration method used for selection of patients. Required information including clinical findings, PE location, fluid appearance, and biochemical characteristics were recorded using a checklist and analyzed via appropriate statistical methods.

***Results:*** A total of 223 patients were included in this study. Congestive heart failure (CHF) was the most common cause of PE (n=67), followed by pulmonary thromboembolism and malignant diseases. PE fluid in all patients with CHF was transudative.

***Conclusion:*** According to the findings of this study, CHF was the most prevalent cause of PE.

## Introduction

Pleural effusion (PE) is one of the most common manifestations of pulmonary diseases that accumulates in pleural space by two major mechanisms: 1. Changes in the hydrostatic and oncotic pressures in situation such as heart failure, nephrosis, liver cirrhosis and, 2. Increasing the permeability of pleural membranes in patients with pleuritis due to various etiologies including infection, cancer and pulmonary embolism.^1^

The prevalence of different etiologies of PE has not been examined on a population level in Iran. Several studies have found tuberculosis as the most common etiology in non-purulent PE in developing countries. In a study performed on patients with non-purulent PE in Qatar, tuberculosis (32.5%), pneumonia (19%), malignant PE (15.5%), and heart failure (13%) were described as the main causes of PE.^2^

A study in Bangladesh reported the tuberculosis as the most common cause of PE followed by parapneumonic effusion, malignancy, nephrotic syndrome, liver cirrhosis, and congestive heart failure (CHF).^3^ The same results were reported from other developing countries and proved tuberculosis as the most common cause of PE.^4^

On the other hand, according to the epidemiologic studies in this regard, CHF, malignancy, and pneumonia are the most common causes of PE in developed countries.^5,6^ A study by Porcel et al revealed that heart failure is one of the most common causes of PE, which accounted for 80% of transudative effusions and more than half of all effusions in patients over 80 years old.^7^

Since the etiologic factors causing PE in different regions can be influenced by the epidemiology of common diseases in that area, this study was conducted to determine the prevalence of etiologic factors in patients referred to a tertiary hospital in the North-West of Iran.

## Materials and Methods

During May 2015 and May 2016, a descriptive cross-sectional study was conducted to determine the epidemiology of various causes of PE in patients referred for paracentesis to pulmonary division of Imam Reza Hospital, a tertiary hospital at Tabriz, North-West of Iran.

Sampling was achieved using complete enumeration method. The enrollment criteria for this study were age more than 15 years and detection of PE as the cause of referral of the patient. On the other hand, patients with incomplete medical records or who refused thoracentesis were excluded from the study. Demographic data, clinical signs and symptoms, and appearance of the pleural fluid was collected using a checklist.

Data were analyzed using the Statistical Package for the Social Sciences version 16 (SPSS Inc., Chicago, IL, USA). Descriptive statistics were used to express the demographic variables. Quantitative variables were reported as mean ± standard deviation and qualitative variable as number and percentage.

## Results

### Demographic data

A total of 231 patients with PE were enrolled in this study. Eight patients excluded due to inadequate data. Finally, 223 patients were evaluated. Of these, 119 were men (53.4%) and 104 were women (46.6%). The mean age of patients was 61.05±16.83 years old, ranging between 24 and 84 years (Table 1).

**Table 1:** The etiology and characteristics of the pleural effusion in the North-West of Iran

**Variables**	**CHF**	**Pneumonia**	**PTE**	**TB**	**Empyema**	**Infl.**	**SLE**	**Self-limited**	**Malignancy**	**Cirrhosis**	**ESRD**	**Nephrotic syndrome**	**Pancreatitis**	**Hypothyroidism**
No. (%)		67 (30.0)	31 (13.9)	35 (15.7)	35 (15.7)	11 (4.9)	10 (4.5)	8 (3.6)	6 (2.7)	5 (2.2)	3 (1.3)	3 (1.3)	3 (1.3)	3 (1.3)	3 (1.3)
Age (y)		76.31	76.31	41.69	60.94	52.0	47.0	38.33	44.25	60.94	55.0	53.64	36.0	53.4	40.67
Gender (%)	M	58	63	26	50	100	100	33	50	51	87	45	33	80	33
	F	42	37	74	50	0	0%	67%	50%	49%	12%	54%	67%	20%	67%
Fluid nature (%)	Ts.	100	0	22	0	0	0	0	0	0	100	100	100	0	100
	Ex.	0	100	77	100	100	100	100	100	100	0	0	0	100	0
Disease course (%)	A	0	100	100	0	100	100	0	100	0	0	0	0	100	0
	SA	0	0	0	40	0	0	100	0	89	0	91	100	0	0
	C	100	0	0	60	0	0	0	0	6	100	9	0	0	100
Collection location (%)	R	38	45	49	40	67	0	0	0	42	100	27	33	0	0
	L	14	45	49	30	33	0	33	50	42	0	54	0	100	0
	B	47	0	2	30	0	100	67	50	14	0	18	67	0	100
Cell count (%)	PMN	71.92	78.38	76.71	29	80	37.5	26.66	40	76.14	53.75	65.45	65	66	71.66

CHF, congestive heart failure; PTE, pulmonary thromboembolism; TB, tuberculosis; Influ, influenza; SLE, systemic lupus erythematosus; ESRD, end-stage renal disease; NS, nephrotic syndrome; HypoT, hypothyroidism; SD, standard deviation; M, male; F, female; Ex, exudate; Ts, transudate; A, acute; SA, subacute; C, chronic; R, right; L, left; B, bilateral; PMN, polymorphonuclear.

### Clinical findings

Of 223 patients, 80 (35.7%) had acute presentation, 55 (24.6%) had subacute presentation, and 88 (39.6%) had chronic presentation of disease. The demographic and pleural fluid characteristics of patients are shown in Table 1. Congestive heart failure (CHF) was the most prevalent cause of PE among patients. A total of 82.0% of them had systolic and others had diastolic heart failure according to echocardiography findings.

The most common clinical presentation of the patients was dyspnea (n=206, 92.8%). Moreover, 58 patients had fever (26.1%), 54 (24.3%) presented with chills, 156 (70.3%) with anorexia, 49 (22.1%) with chest pain, 70 (31.5%) with weight loss, and 11 (5%) with excessive sweating.

On the other hand, 78 (35.6%) patients had left side PE, 89 (40.6%) patients had right side PE and 52 (23.7%) of them had bilateral PE. Also, 2 (5.71%) PTE patients had mild pericardial effusion.

### Laboratory findings

**Figure 1:**
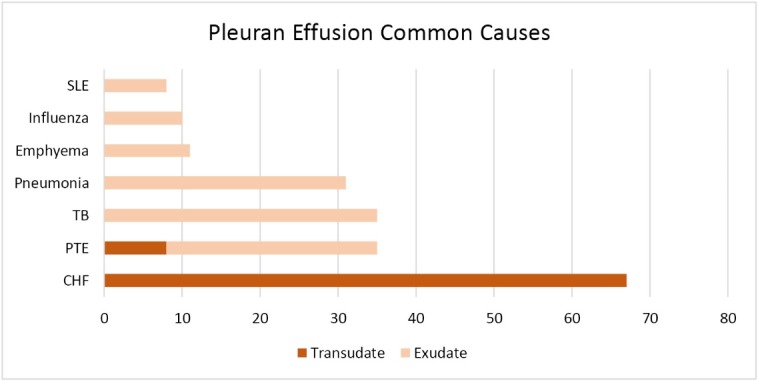
Common etiologies of exudate and transudate pleural effusion in the North-West of Iran. CHF, chronic heart failure; PTE, pulmonary thromboembolism; TB, tuberculosis; SLE, systemic lupus erythematosus.

The pleural fluid appearance was clear yellow in 73 (36.3%) cases, yellow amber in 98 (48.8%), dark-brown in 7 (3.5%), milky in 3 (1.5%), and bloody in 17 (8.5%) patients. Also, three patients had hemothorax. In 76 (38.8%) cases, the nature of the fluid was transudative, and in 120 (61.2%) patients it was exudative (Figure 1). The fluid was loculated in 49 (22.1%) patients. Imaging studies revealed no concurrent lung parenchymal lesion in 84 (37.7%) patients.

Mean hemoglobin levels were 12.54±2.10 g/dL ranging between 7.5 g/dL and 17.10 g/dL. Also, the mean white blood cell count was 9786/mL ranging between 1200/mL and 50 000/mL.

## Discussion

The findings of the present study showed that PE has a higher incidence in the elderly because the mean age of the patients was 65 years. This finding is consistent with the results of other studies in this regard.^6,8,9^ However, our findings are not consistent with those of the Qatar study, where the highest prevalence of PE was approximately 40 years and tuberculosis was the cause of considerable number of PE, especially among youngers.^2^

CHF was the leading cause of PE in our study population. This finding is in contrast to the findings of other studies conducted in the Persian Gulf. In a study conducted in Qatar, tuberculosis was the most common cause of PE in patients admitted to hospitals. The prevalence of tuberculosis-induced PE is considerably higher than other etiologies in the Qatar study.^2^ Similarly, tuberculosis was the most prevalent etiologic cause of PE in studies conducted in Saudi Arabia, India, Malaysia, Lebanon, Iraq and Ghana.^4,8-12^

Liam et al found that the most common causative disease of exudative PE was tuberculosis (44.1%), followed by malignancy (29.6%), the most common of which was lung cancer.^9^ Al-Alusi described a series of 100 patients with PE in Iraq and found that tuberculosis was the most common cause of PE in Iraq followed by cancer.^12^ In another study conducted in Saudi Arabia, tuberculosis was the most common single cause of PE among young patients and patients with Indian and Yemeni nationality.^8^

Another study performed in Ghana showed tuberculosis as the leading cause of PE in adults with HIV infection.^10^ In a comprehensive study performed in Spain, a total of 3077 patients who underwent diagnostic thoracocentesis were evaluated. This study showed that cancer (27%) was the single most common cause of PE among studied patients, followed by heart failure (21%), pneumonia (19%), tuberculosis (9%), abdominal surgery (4%), pericardial diseases (4%) and cirrhosis (3%).^13^ This discrepancy between Ghana and Spain may justified by the different in developing level of two countries.

In a rather old study conducted in Iran, the etiological epidemiology of PE was consistent with the findings of the present study and attributed the high prevalence of heart disease in this country. The second most common cause of PE was tuberculosis.^14^ Of note, the incidence of tuberculosis-related PE in our region was lower compared to Isfahan. The overall decrease in the prevalence of tuberculosis and also early diagnosis and treatment in Iran can play a role in decreasing incidence of tuberculosis-related PE.

Considering the descriptive nature of the study as well as the way in which data have been collected (referral to a tertiary center), the findings of this study cannot be generalized to the community level and all patients with PE. Indeed, some PEs may be small and do not have significant symptoms, so did not referred to a tertiary centre.

Also, the results of this study revealed that the prevalence of PE due to empyema was very lower than other etiologies. This could be the result of early diagnosis and appropriate therapy of pneumonia. Finally, a number of malignancy-related PEs may not be referred to pulmonary clinics and investigated by the oncologist that may decrease the malignancy-related PE in the study population.

The low prevalence of tuberculosis-related PE in this study compared to studies from other countries in the Persian Gulf area may be due to successful screening, diagnosis and treatment programs in Iran and also lower number of emigrants from countries with high incidence of tuberculosis.

## Conclusion

The present study showed that the most prevalent cause of PE in North-Western Iran is CHF, followed by pulmonary thromboembolism and malignancies.

## Competing interests

None.

## Ethical approval

The study was approved by the Regional Ethics Committee of Tabriz University of Medical Sciences (Approval ID: TBZMED.REC.1394.1205) and informed consent obtained from all patients.

## Funding

The authors declare that they have no financial interests to disclose.

## Acknowledgments

The authors are grateful to patients for their contribution to this study.
